# FDA approved antibacterial drugs: 2018-2019

**DOI:** 10.15190/d.2019.15

**Published:** 2019-12-31

**Authors:** Stefan Andrei, Gabriela Droc, Gabriel Stefan

**Affiliations:** Department of Anesthesia and Intensive Care, Fundeni Clinical Institute, Bucharest, Romania; Carol Davila University of Medicine and Pharmacy, Bucharest, Romania; Université Paris Sud XI, Faculté de Médecine, Le Kremlin-Bicêtre, France; Dr. Davila Teaching Hospital of Nephrology, Bucharest, Romania

**Keywords:** FDA approved drugs, Plazomicin, Eravacycline, Sarecycline, Omadacycline. Rifamycin, Imipenem, Cilastatin and Relebactam, Pretomanid, Lefamulin, Cefiderocol, 2018, 2019.

## Abstract

Bacterial resistance to existent antibiotherapy is a perpetual internationally-recognized problem. Year after year, there is a continuous need for novel antibacterial drugs and this research and development efforts recently resulted in few new drugs or combination of drugs proposed for the use into the clinic. This review focuses on the novel US FDA approved antibacterial agents in the last two years (2018-2019). Plazomicin, eravacycline, sarecycline, omadacycline, rifamycin (2018) and imipenem, cilastatin and relebactam combination, pretomanid, lefamulin, cefiderocol (2019) are new therapeutic options. Plazomicin aminoglycoside antibiotic targets Enterobacteriaceae infections, being mainly used for the complicated urinary tract infections. The fully synthetic fluorocycline eravacycline gained approval for the complicated intra-abdominal infections. The tetracycline-derived antibiotic sarecycline might be a useful strategy for the management of non-nodular moderate to severe acne, while the other tetracycline-derived antibiotic approved, omadacycline, may be used for the patients with acute bacterial skin and skin structure infections and community-acquired bacterial pneumonia. The already-known RNA-synthesis suppressor rifamycin is now also approved for noninvasive Escherichia Coli-caused travelers' diarrhea. Two combinatorial strategies were approved for complicated urinary tract infections, complicated intra-abdominal infections (imipenem, cilastatin and relebactam) and lung tuberculosis (pretomanid in combination with bedaquiline and linezolid). Lefamulin is a semisynthetic pleuromutilin antibiotic for community-acquired bacterial pneumonia, while cefiderocol, a cephalosporin antibiotic is the last antibacterial drug approved in 2019, for the use in complicated urinary tract infections. Despite of these new developments, there is an ongoing need and urgency to develop novel antibiotic strategies and drugs to overrun the bacterial resistance to antibiotics.

## 1. Introduction

The run to overcome the rapid bacterial resistance started with the initial use of antibiotics. However, after decades of struggling in research and in clinical practice, this run is rather a marathon than a sprint.**

Despite sustained efforts, the physicians are continuously confronting worldwide with the threat of bacterial resistance^1-3^. The burden on public health contributed to the creation and implementation of strategies on rational antibiotic use and on limiting the spread of resistant bacteria, the so called antibiotic stewardship^4-7^.**

Other strategy in this direction is to optimize the existing pharmaceutical arsenal, through novel combinations and new indications^8^. However, the number and efficiency of these drugs is far from covering all the existing needs and to fully combat the highly adaptive bacterial microorganisms. Moreover, pan-resistant bacteria emergence has been already described^9,10^. Other evidences further consider the non-negligible role of environmental and agriculture-related factors^11,12^.

Antibiotic stewardship has proved its efficiency, but it has its own limits and challenges^13-15^. However, the quest for new efficient molecules have to continue, remaining a pillar of anti-multidrug resistant germs strategy^16^.

We briefly review here the novel antibacterial agents approved by the United States Food and Drug Administration (US FDA) during the past 2 years (2018 and 2019) with the hope to further encourage the scientific community in continuing the development of new therapeutic agents for targeting the resistance of bacteria. The recently approved antibacterial drugs and drug combinations were identified using FDA’s website (https://www. accessdata.fda.gov; www.fda.gov) and Center Watch’s site (https://www.centerwatch.com/drug-information /fda-approved-drugs/).

**Figure 1:**
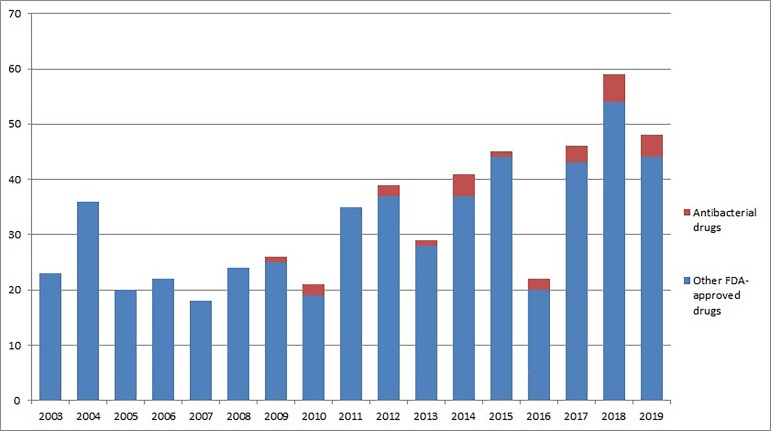
Novel FDA-approved antibacterial and non-bacterial drugs by year (last 17 years) (updated and modified from^17^)

We reviewed the total number of drugs and drug combinations (blue) and number of antibacterial drugs and drug combinations (red) approved by the US FDA in the past 17 years (2003-2019) for each individual year**(**Figure 1,** updated and modified from^17^). There is a clear upward trend in terms of the number of antibacterial drugs approved by year.

To the date of this review, 9 antibacterial drugs or combination of drugs were approved for 2018 and 2019, from a total of 107 introduced molecules. In 2018, the 5 approved antibacterial drugs or combinations of drugs represented 8.4% of the total of 59 new drugs, while in 2019 the 4 new single or combination of antibacterial drugs represented 8.33% of the 48 approved molecules. This represents a significant increase from the previous years (**Figure 1**), since the number of antibacterial drugs or regimens in the past two years has doubled as compared to the two previous years.

We identified 9 novel FDA approved antibacterial drugs: plazomicin aminoglycoside antibiotic, the fully synthetic fluorocycline eravacycline, tetracycline-derived antibiotics sarecycline and omadacycline, RNA synthesis suppressor rifamycin, two combinatorial strategies (imipenem (carbapenem antibiotic), cilastatin and relebactam and pretomanid (nitroimidazole) in combination with bedaquiline and linezolid), the semisynthetic pleuromutilin antibiotic lefamulin and cefiderocol, a cephalosporin antibiotic (**Table 1 & Table 2**). These drugs are briefly discussed by FDA-approval year in the next section.

**Table 1:** Main characteristics of the described antibacterial drugs FDA approved in 2018^18-35^ Food and Drug Administration (FDA), European Medicines Agency (EMA), complicated urinary tract infections (cUTIs), complicated intra-abdominal infections (cIAIs), community-acquired bacterial pneumonia (CABP), acute bacterial skin and skin structure infections (ABSSI), intravenous (IV), oral administration/per oral (PO), hours (h); *doses and duration have to be verified in the most recent prescriptions reglementations, according to the local laws before administration.*

NAME (generic/brand/ class)	Approval status	Indication	Administration	Dose and duration
Plazomicin (Zemdri)/ aminoglycoside antibiotic COMPANY: Achaogen, Inc. (CA, USA)	FDA: approved in June 2018 EMA: application submitted (2018)	cUTIs; Enterobacteriaceae infections	IV infusion, every 24 hours for 4-7 days.	A. Dosage regimen (adults with CrCl>60ml/min): 15 mg/kg every 24 hours B. Dosage regimen (adults with CrCl>30 <60 ml/min): 10 mg/kg every 24h C. Dosage regimen (adults with CrCl>15 <30 ml/min): 10 mg/kg every 48h
Eravacycline (Xerava) / fully synthetic fluorocycline COMPANY: Tetraphase Pharmaceutical (MA, USA)	FDA: approved in August 2018 EMA: approved in September 2018	cIAI	IV 60 min infusion, given once every 12 hours for a total of 4 to 14 days; dose is patient’s weight dependent	Adult patients (≥18 years of age) with cIAI: administer 1mg/kg, every 12h, by IV infusion (~ 60min); recommended duration of treatment is 4 to 14 days
Sarecycline (Seysara) / tetracycline-derived antibiotic COMPANY: Paratek Pharmaceuticals (MA, USA) and Allergan plc (USA) – acquired by Almirall SA (Spain)	FDA: approved in October 2018 EMA: not yet approved	non-nodular moderate to severe acne	PO administration with food	A. Adult <54 kg: 60 mg PO every Day 55-84 kg: 100 mg PO every Day 85-136 kg: 150 mg PO every Day B. Children ≥9 years 33-54 kg: 60 mg PO every Day 55-84 kg: 100 mg PO every Day 85-136 kg: 150 mg PO every Day If improvement after 12 weeks not observed, reassess treatment
Omadacycline (Nuzyra) / aminomethylcycline antibiotic, tetracycline class (inhibits 30S ribosomal subunit) COMPANY: Paratek Pharmaceuticals (MA, USA)	FDA: approved in October 2018 EMA: not yet approved	CABP, ABSSSI	Both once-daily IV and PO formulations	A. For patients with CABP, the loading dose on day 1 is 200 mg by IV infusion over 60 minutes, or 100 mg by IV infusion over 30 minutes, given twice; the maintenance dose is 100 mg by IV infusion over 30 minutes once daily, or 300 mg PO daily for a total of 7 to 14 days. B. For patients with ABSSSI, the loading dose on day 1 is 200 mg by IV infusion over 60 minutes; or 100 mg by IV infusion over 30 minutes, given twice; or, on days 1 and 2, 450 mg orally once daily. The maintenance dose is 100 mg by IV infusion over 30 minutes once daily, or 300 mg PO daily, for a total duration of 7 to 14 days
Rifamycin (Aemcolo) / bactericidal; inhibit bacterial DNA-dependent RNA polymerase, suppressing RNA synthesis COMPANY: Cosmo Pharmaceuticals (Ireland)	FDA: approved in November 2018 EMA: not yet approved	Travelers' diarrhea (noninvasive strains (E. coli)	PO administration	388 mg (2 tablets) PO twice a day x 3 days

**Table 2:** Main characteristics of the described antibacterial drugs FDA approved in 2019^36-49^ Food and Drug Administration (FDA), European Medicines Agency (EMA), complicated urinary tract infections (cUTIs), complicated intra-abdominal infections (cIAIs), community-acquired bacterial pneumonia (CABP), intravenous (IV), oral administration/per oral (PO), hours (h); *doses and duration have to be verified in the most recent prescriptions reglementations, according to the local laws before administration*

NAME (generic/brand/ class)	Approval status	Indication	Administration	Dose and duration
Imipenem, cilastatin, relebactam (Recarbrio) COMPANY: Merck & Co (NJ, USA)	FDA: approved in July 2019 EMA: not yet approved	cUTI, cIAI	IV infusion	Injection, powder for reconstitution 500mg/500mg/250mg per vial (ie, 1.25g/vial) A. Urinary Tract Infection: 1.25 g IV every 6h x 4-14 days B. Intra-abdominal Infections 1.25 g IV every 6h x 4-14 days
Pretomanid / nitroimidazole, a class of novel anti-bacterial agents, in combination with bedaquiline and linezolid COMPANY: TB Alliance (NY, USA & South Africa; non-profit)	FDA: approved in August 2019 EMA: not yet approved	Drug-resistant TB (lung tuberculosis)	PO, one tablet (200ml, adult) taken once a day for 26 weeks	Pretomanid 200 mg PO/day x 26 weeks Bedaquiline 400 mg PO/day x 2 weeks, Then, 200 mg 3x/week with at least 48 h between doses for x 24 weeks (total of 26 weeks) Linezolid 1200 mg PO/day for 26 weeks
Lefamulin (Xenleta) / semisynthetic pleuromutilin antibiotic COMPANY: Nabriva Therapeutics (Ireland)	FDA: approved in August 2019 EMA: not yet approved	CABP	IV and PO treatment	600 mg orally every 12 hours for 5 days or 150 mg infused IV over 60 minutes every 12 hours for 5-7 days; IV: -Mild or moderate liver dysfunction (Child-Pugh A or B): No adjustment recommended. -Severe liver dysfunction (Child-Pugh C): 150 mg IV every 24 hours Oral: -Mild liver dysfunction (Child-Pugh A): No adjustment recommended. -Moderate or severe liver dysfunction (Child-Pugh B or C): Not recommended.
Cefiderocol (Fetroja) / cephalosporin antibacterial COMPANY: Shionogi & Co., Ltd. (Japan)	FDA: approved in November 2019 EMA: not yet approved	cUTI	IV	2 gram IV every 8h for 7-14 days

## 2. FDA approved antibacterial drugs (2018-2019)

### ** *2.1 Plazomicin*

Plazomicin sulfate (Zemdri) is a semisynthetic aminoglycoside bactericidal antibiotic drug, acting in asimilar manner to other aminoglycosides, by suppressing the 30S bacterial ribosomal subunit.**Noteworthy, while other aminoglycosides can be inactivated by aminoglycoside-modifying enzymes, plazomicin is resistant to the action of these enzymes^18^.

Plazomicin was approved by FDA in June 2018, for targeting the infections with Gram-negative aerobic bacteria in the complicated urinary tract infections (cUTIs). Application was submitted for review by European Medicine Agency (EMA) in June 2018.

Plazomicin is administered intravenously (IV) every 24 hours for 4-7 days; it is primarily active against Gram-negative aerobic bacteria (e.g. *Enterobacteriaceae family*), to be used in patients over 18 years of age with cUTIs (including pyelonephritis), caused by susceptible *Escherichia coli*, *Klebsiella pneumoniae*, *Proteus mirabilis* or *Enterobacter cloacae^18,19^*.

Plazomicin is supplied as single-dose vials in an amount of 500 mg/10 mL plazomicin base^19^. Administration recommendations can be found in **Table 1** and its dosage is personalized based on the renal function and/or therapeutic monitoring of the drug, if available^19^. Plazomicin might be of value in patients that have resistance to their primary treatment options or who are allergic to beta-lactam antibiotics.

The most important adverse events reported with plazomicin are: nephrotoxicity (but lower incidence of nephrotoxicity than colistin), diarrhea, hypertension, headache, nausea, vomiting, hypotension^20^.

Plazomicin showed to be non-inferior to meropenem within the EPIC non-inferiority trial in treatment of cUTIs and even demonstrated superior microbiological eradication (81.7% versus 70.1%; 95% confidence interval (CI) 2.7-25.7)^21^. Plazomicin-based combinations also demonstrated decreased disease-complications and mortality when compared to colistin-based combination in the CARE trial (23.5% versus 50%; 90% CI -0.7 to 51.2)^20^**.**

### *2.2 Eravacycline*

Eravacycline dihydrochloride (Xerava) is a fully synthetic bacteriostatic fluorocycline and a tetracycline-class antibacterial agent that binds bacterial 30S ribosomal subunit*. *Compared to other tetracyclines, it has two structural substitutions which makes the drug working on certain strains of Gram-positive and Gram-negative bacteria that usually have tetracycline-specific resistance mechanisms. Noteworthy, eravacycline can be used (at least in cell culture) to target *Enterobacteriaceae*, in the presence of certain beta-lactamases^18,22^.

Eravacycline was approved by FDA in August 2018 and by EMA in September 2018, being indicated in the complicated intra-abdominal infections^22-24^.

Eravacycline is administered IV in 60 min infusions, given once every 12 hours for a total of 4 to 14 days; dose is patient’s weight dependent (1mg/kg) and it is used in persons over 18 years of age with complicated intra-abdominal infections (cIAI) caused by susceptible microorganisms identified in the prescribing information. This is the only indication of use for eravacycline at this moment, although it may be approved for other applications in the future, similar to other tetracyclines^18^.

The most important adverse event reported with eravacycline in clinical trials and sometimes a cause of the treatment discontinuation is the gastrointestinal (GI) upset. Other noteworthy adverse events that can appear are infusion site reactions, nausea, and vomiting^18,22^.

Eravacycline was compared with ertapenem and meropenem for the treatment of cIAIs in 2 non-inferiority trials (IGNITE1 and IGNITE4), with similar clinical response rates^25,26^.

### *2.3* *Sarecycline*

Sarecycline hydrochloride (Seysara) is a new, narrow-spectrum tetracycline derivative. It proves antibacterial activity against skin and soft tissue pathogens, including the *Cutibacterium acnes* (anaerobic Gram-positive bacterium related to acne development). Similar to other tetracyclines, it possesses anti-inflammatory effects. However, it has some specific properties comparing to other tetracyclines: it seems to affect the intestinal flora less; it shows a lower rate of resistance to tetracycline-resistant *Staphylococcus aureus*, as well as erythromycin-resistant and clindamycin-resistant *Cutibacterium acnes* strains^27^. Sarecycline has significant effects on inflammatory lesions. However, it was also noted to show statistically significant effects on noninflammatory acneiform lesions at certain time points^28^.

Sarecycline was approved by FDA in October 2018, for the treatment of non-nodular moderate to severe acne. Application was also submitted for review by European Medicine Agency (EMA) in October 2018.

The drug is administrated as 1.5 mg/kg/day orally with food, in patients aged 9 and older, as a once daily antibiotic with statistically significant improvement seen as early as 3^rd^ week. More detailed information about its administration can be found in **Table 1**.

In clinical trials comparing with placebo evaluating the adverse effects, nausea was reported in 3.1% of the patients treated with sarecycline versus 2.0% in patients treated with placebo; the other adverse reactions reported were found in less than 1% of female subjects treated with sarecycline: vulvovaginal mycotic infection (0.8%) and vulvovaginal candidiasis (0.6%)^29^.

### *2.4 Omadacycline*

Omadacycline (Nuzyra) is an aminomethylcycline antibiotic belonging to the tetracycline class. It inhibits 30S bacterial ribosomal subunit. Compared to other tetracycline antibiotics, omadacycline has structural modifications at the C9 and C7 positions of the core tetracycline rings, enabling ribosomal protection mechanisms and stability in the efflux pump related to resistance to the tetracycline antibiotics^30^.

Omadacycline was approved by FDA in October 2018 for the treatment of community-acquired bacterial pneumonia (CABP) and acute bacterial skin and skin structure infections (ABSSI) and it is not yet approved by the EMA. It can be administered once-daily in both IV and PO formulations (**Table 1**).

Adverse events noted Omadacycline has warnings associated with tetracycline-class antibiotics, including: tooth discoloration, enamel hypoplasia and inhibition of bone growth in late pregnancy, infancy, or childhood up to 8 years of age. The most common adverse reactions (incidence ≥2%) seen in clinical trials of omadacycline are: nausea, vomiting, infusion site reactions, alanine aminotransferase increased, aspartate aminotransferase increased, gamma-glutamyl transferase increased, hypertension, headache, diarrhea, insomnia, and constipation. Omadacycline has only been studied in patients 18 years of age or older. Omadacycline’s affinity for muscarinic M2 receptors induces a transient heart rate increases and it has no effect on the QT interval^31^.

A meta-analysis of randomized controlled trials revealed that the clinical efficacy of omadacycline is not inferior to that of competitor drugs in the treatment of acute bacterial infections in adult patients^32^.

### *2.5* *Rifamycin*

Rifamycin (Aemcolo) is a**bactericidal minimally absorbed antibiotic that inhibits bacterial DNA-dependent RNA polymerase, by suppressing RNA synthesis. It is the first antibiotic engineered with Cosmo Pharmaceuticals’ Multi Matrix Technology, that enables colonic release of the active compound^33^.

It was approved by FDA in November 2018 for the treatment of the noninvasive strains of *Escherichia coli* causing travelers' diarrhea, in an orally dose of 388 mg (2 tablets) twice a day for 3 days but not when diarrhea is complicated by fever and/or bloody stools^34.^

The most important adverse reactions observed during the clinical trials are constipation (3.5%), headache (3.3%), abdominal pain (0.5%) & pyrexia (0.3%) with 1% of the patients discontinuing the treatment^33,34^.

In a randomized double-blind phase 3 study (ERASE), Rifamycin SV-MMX was found to be equally effective as ciprofloxacin and to not induce resistance in bacteria for the treatment of travellers' diarrhea^35^.

### *2.6 Imipenem, cilastatin and relebactam (Recarbrio)*

Recarbrio is a regimen comprising of imipenem, a penem antibacterial, cilastatin, an inhibitor of the renal dehydropeptidase and relebactam, a beta-lactamase inhibitor^36^.

It was approved by FDA in July 2019 for the treatment of cUTIs (including pyelonephritis) and cIAI in patients 18 years of age and older who have limited or no other treatment options available. Susceptible bacteria are Gram-negative microorganisms such as *Enterobacter cloacae*, *Escherichia coli*, *Klebsiella pneumoniae*, *aerogenes* and *oxytoca*, *Pseudomonas aeruginosa*, some strains of *Bacteroides* and other susceptible bacteria^37^.

It is administered as a 30 min IV infusion: 500mg/500mg/250mg per vial (1.25g/vial): 1.25 g IV every 6h x 4-14 days (for cUTI) and 1.25 g IV every 6h x 4-14 days (for cIAI)^38^.

Adverse events observed with this triple combination include, but are not limited to: diarrhea, nausea, headache, vomiting, increase in transaminase, phlebitis/infusion site reactions, pyrexia, hypertension^36^.

### *2.7 Pretomanid*

Pretomanid is a nitroimidazole, a class of novel anti-bacterial agents. It was approved in August 2019 by the FDA to be used in combination with bedaquiline, which targets the adenosine triphosphate (ATP) synthase enzyme of the TB mycobacteria and linezolid, a synthetic antibiotic, the first of the oxazolidinone class, for the treatment of drug-resistant TB (lung tuberculosis). It is not yet approved by the EMA.

It is orally administered, one tablet (200 ml) taken once a day for 26 weeks for adults. Specifically, pretomanid 200 mg PO/day x 26 weeks, bedaquiline 400 mg PO/day x 2 weeks, then, 200 mg 3x/week with at least 48 h between doses for x 24 weeks (total of 26 weeks), and linezolid 1200 mg PO/day for 26 weeks^39^.

Adverse events reported with pretomanid include numbness and tingling of extremities, acne, anemia, nausea, vomiting, headache, increased transaminases, excess amylase in the blood, indigestion, decreased appetite, abdominal pain, rash, itching, sharp chest pain during breathing, increased gamma-glutamyl transferases, lower respiratory tract infection, cough, coughing up blood, back pain, visual impairment, low blood sugar (hypoglycemia), abnormal weight loss, diarrhea^40^.

The early efficacy reported in a recently published trial, showed that the pretomanid-containing regimens had a more significant early bactericidal activity than classical a HRZE regimen^41^. However, more research is needed before pretomanid can be validated as a promising therapy in tuberculosis^42^.

### *2.8 Lefamulin*

Lefamulin (Xenleta) is a semisynthetic pleuromutilin antibiotic that binds to the peptidyl transferase center of the 50S bacterial ribosomal subunit, inhibiting protein synthesis within bacteria^43^.

It was approved by FDA in August 2019 for the treatment of CABP. Lefamulin is not yet approved by EMA.

It can be administered either as an IV infusion or PO. It is used as 600 mg orally every 12 hours for 5 days or 150 mg infused IV over 60 minutes every 12 hours for 5-7 days. For additional information please consult **Table 2**.

As adverse events, lefamulin can prolong the QT interval (increased risk in patients with renal failure or hepatic dysfunction), produces infusion-site reactions, diarrhea, hepatic enzyme elevations, nausea, hypokalemia, insomnia, and headache. There are multiple other drugs interactions and it should not be used in pregnant women^43^.

FDA approval of lefamulin was based on the results of 2 randomized, controlled double-blind, noninferiority trials called LEAP 1 and LEAP 2, which enrolled 1289 adults^44,45^. Results of these trials demonstrated that lefamulin was as effective as moxifloxacin in the treatment of the community-acquired bacterial pneumonia^43^.

### *2.9 Cefiderocol*

Cefiderocol (Fetroja) is a siderophore cephalosporin antibacterial drug that has been developed to fight a wide range of bacterial pathogens, such as the β-lactam-resistant and carbapenem-resistant orga-nisms^46^.

It was approved in November 2019 for the treatment of complicated urinary tract infections. It is not yet approved by EMA.

It is administered as an IV infusion, 2 gram every 8h, for 7-14 days. Cefiderocol targets a wide range of clinically relevant gram-negative bacteria, including but not limited to the *Enterobacteriaceae spp*, such as *Enterobacter spp*, *Klebsiella spp, Proteus spp, Vibrio spp, Yersinia spp, Serratia marcescens, Shigella flexneri, Salmonella spp* and also nonfermenting bacterial species such as *Acinetobacter and Pseudomonas^46-48^*.

As adverse effects, cefiderocol is well tolerated, with minor reports of gastrointestinal and phlebitis. This side effect profile is similar to the profile of other cephalosporin drugs^49^.

Clinical trials have shown that Cefiderocol's activity against bacteria non-susceptible for meropenem and *Klebsiella pneumoniae* carbapenemase-producing *Enterobacteriales* is similar or even superior to ceftazidime-avibactam. Cefiderocol is also more potent than meropenem and ceftazidime-avibactam in targeting *Pseudomonas aeruginosa* (against all resistance phenotypes) and *Stenotrophomonas maltophilia^49^*.

## 3. Promising antimicrobials under investigation

Comparing with the last decade, we have the impression of an acceleration of antibiotic succeeded approval. However, this do not meet the urgency of WHO and United Nations calls for action^50^.

At the end of 2019, a total of 42 new antibiotics or new combinations are in different stages of clinical development globally, a certain number being already approved by FDA^51^. Iclaprim stands out by the high possibility of a soon FDA-approval. This molecule with diaminopyrimidine structure acts by inhibiting bacterial dihydrofolate reductase and it has been successfully tested in a phase 3 randomized controlled trial (REVIVE-1), showing to be non-inferior to vancomycin in treating ABSSI^52^. By its novel mechanism of action, lack of nephrotoxicity and capacity to suppress bacterial exotoxins, iclaprim might become an interesting player in treating resistant Gram positive germs^53^.

Gram-negative bacteria are protected by a double membrane envelope, which forms a highly efficient barrier to antibiotics. The external membrane contains lipopolysaccharide molecules in the outer layer and integral outer-membrane proteins (OMPs). OMPs are folded into the membrane by a protein complex called the β-barrel assembly machine (BAM), which have a central component called BamA accessible from the bacterial surface. Three recent studies report new antibiotics that seem to target BamA - therefore inhibiting the normal OMP folding that is necessary for bacterial survival - darobactin, murepavadin analogues and MRL-494.

Darobactin was efficient against multiple Gram-negative bacteria (*in vitro *and in infected mice): polymyxin-resistant *Pseudomonas aeruginosa*, β-lactam-resistant *Klebsiella pneumoniae* and *Escherichia coli^54^. *

Murepavadin analogues, obtained after linking the murepavadin molecule with the lipopolysaccharide binding portion of polymyxin B, displayed antibiotic activity against *Klebsiella pneumoniae, Pseudomonas aeruginosa, Escherichia coli^55^.*

MRL-494, a newly identified compound, had similar antibiotic effectiveness *in vitro *against both wild-type *Escherichia coli*and a mutant defective in outer-membrane integrity and efflux mechanisms, suggesting that this antibiotic might not need to breach the membrane to exert its activity. However, MRL-494 efficiency remains to be tested in animal models^56^.

Small bacterial toxins can act as antimicrobial peptides. Reducing their toxicity to human cells and retaining their antibiotic activity can open new perspectives in antibiotics development.

Recently, Nicolas et al. synthesized 4 cyclic heptapseudopeptide biomimetics, which reproduce a section of a *Staphylococcus aureus *toxin, PepA1^57^. Two of the studied peptides were effective against methicillin-resistant Staphylococcus aureus**in mild and severe sepsis mouse models without displaying toxicity on human erythrocytes and kidney cells, zebrafish embryos, and mice. Moreover, efficacy was also proved against *Pseudomonas aeruginosa *and *MRSA* in a mouse skin infection model. Notably, these novel compounds did not lead to resistance after serial passages for 2 weeks and 4- or 6-days’ exposure in mice.

The ability of unnatural amino acids to strengthen dynamic association with bacterial lipid bilayers and to induce membrane permeability can explain the antibiotic effect of the heptapseudopeptides^57^.

Delafloxacin, a new fluoroquinolone already FDA approved for the treatment of acute bacterial skin and skin structure infections, is currently the only antibiotic with *in vitro* activity against methicillin-resistant *Staphylococcus aureus* and *Pseudomonas aeruginosa^17,58^*. Therefore, delafloxacin efficiency in comunity acquired pneumonia has been evaluated in recently completed Phase 3 study (NCT02679573).

Ceftobiprole medocaril, an anti-*MRSA* cephalosporin indicated for the treatment of complicated skin infections and community/hospital acquired pneumonia, it is still unapproved by the FDA, but currently used in some European countries and in Canada^59^.

The new lipoglycopeptide dalbavancin - FDA approved for acute bacterial skin and skin structure infections - has a high microbiological activity against staphylococci and enterococci. Moreover, dalbavancin has long half-life (up to 250 hours), which makes it a suitable option for a more rapid hospital discharge. However, a Phase II study which aimed to evaluate the effectiveness of dalbavancin in patients with blood stream infections or infective endocarditis was stopped early due to economic reasons (NCT03148756) and data are insufficient to support its use in this setting^60,61^.

## 4. Conclusion

There is a significant need for novel antibacterial drugs and this research and development efforts recently resulted in few new drugs or combination of drugs proposed for use into the clinic. There is a significant increase in the number of the new FDA approved drugs in the past 2 years compared to the previous years, since the number of antibacterial drugs or regimens in the past 2 years (9 antibacterial agents) is almost double the number of the ones from any 2 previous years, within the past 17 years.

The novel US FDA approved antibacterial agents in the last two years (2018-2019): plazomicin, eravacycline, sarecycline, omadacycline, rifamycin (2018) and imipenem, cilastatin and relebactam combination, pretomanid, lefamulin, cefiderocol (2019) are new players in the field of resistant bacteria treatment for specific indications. However, the number and efficiency of these new drugs is far from covering all the existing needs, to fully combat highly adaptive microorganisms. Thus, there is a real need and urgency to develop novel antibiotic strategies and drugs to overcome the bacterial resistance to antibiotics.

Through this review, we aim to further encourage the scientific community to continue the development of new therapeutic agents for targeting bacterial resistance.
